# Minutes to midnight: Turning back the Doomsday Clock through neglected disease vaccine diplomacy

**DOI:** 10.1371/journal.pntd.0006676

**Published:** 2018-09-20

**Authors:** Peter J. Hotez

**Affiliations:** 1 Texas Children’s Hospital Center for Vaccine Development, Departments of Pediatrics, National School of Tropical Medicine, Baylor College of Medicine, Houston, Texas, United States of America; 2 Texas Children’s Hospital Center for Vaccine Development, Department of Molecular Virology and Microbiology, National School of Tropical Medicine, Baylor College of Medicine, Houston, Texas, United States of America; 3 Department of Biology, Baylor University, Waco, Texas, United States of America; 4 James A Baker III Institute of Public Policy, Rice University, Houston, Texas, United States of America; 5 Scowcroft Institute of International Affairs, Texas A&M University, College Station, Texas, United States of America; McGill University, CANADA

New information reveals that the 10 nations currently producing nuclear weapons also suffer with approximately one-half of the world’s disease burden from several neglected diseases, including intestinal helminth infections, dengue, and measles.

In the aftermath of World War II and the bombings of Hiroshima and Nagasaki, the Science and Security Board of the Bulletin of the Atomic Scientists established the Doomsday Clock, meant to symbolize the time we have left until global nuclear annihilation strikes at midnight [1]. As of 2018, we have reached two minutes until midnight, the closest we have ever gotten since 1953 when the United States detonated its first hydrogen bomb [1].

**Fig 1:**
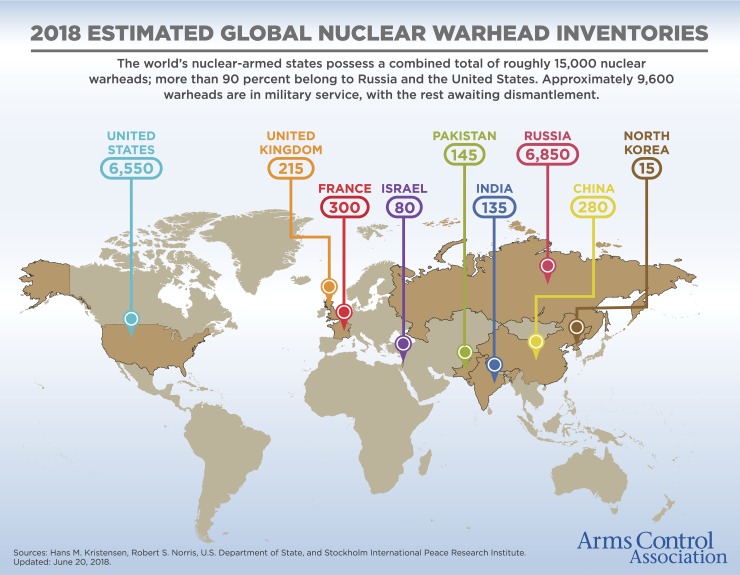
2018 Estimated global nuclear warhead inventories (updated June 20, 2018). https://www.armscontrol.org/factsheets/Nuclearweaponswhohaswhat. *Reprinted with permission from The Arms Control Association*.

Today, the Arms Control Association identifies nine nations—United States, United Kingdom, France, Israel, Pakistan, India, Russia, China, and North Korea—hosting nuclear warhead inventories [2] (Fig 1). Together, these countries account for almost 15,000 nuclear warheads. In addition, while Iran cannot be considered yet to be a nuclear weapons nation, according to the Nuclear Threat Initiative (NTI), it has an advanced nuclear program, including substantial uranium enrichment capabilities [3].

The 10 current nuclear nations also stand out because they are simultaneously affected by neglected diseases. Shown in Table 1 is an assessment of five neglected diseases, including four neglected tropical diseases (NTDs), among the 10 countries, as determined by the Global Burden of Disease Study (GBD) 2016 [4]. It finds that the nuclear weapons states account for more than one-half of the world’s incident measles and dengue cases as well as more than 40% of the global prevalence of intestinal helminth infections (i.e., ascariasis, trichuriasis, and hookworm infection) [4]. GBD 2016 further finds that the 10 nations together also account for a significant percentage of the global incidence of cutaneous leishmaniasis and cystic echinococcosis and likely other NTDs [4].

**Table 1:** Neglected diseases among the 10 nuclear weapons nations.

Country	Nuclear Warhead Inventories^a^	Incidence of Dengue^b^	Incidence of Measles^b^	Incidence of Cutaneous Leishmaniasis^b^	Incidence of Cystic Echinococcosis^b^	Prevalence of Intestinal Nematode^b^ Infections
Russia	6,850	0	853	Endemic^c^	3,337	Endemic^c^
United States	6,550	23,448	668	5	281	Endemic^c^
France	300	0	157	4	55	0
China	280	2.6 million	133,465	15	4,974	232.5 million
United Kingdom	215	0	90	0	33	0
Pakistan	145	2.6 million	63,987	13,026	2,880	30.6 million
India	135	53.2 million	4,441,899	396	24,929	364.3 million
Israel Palestine	80	0 0	81 12,752	318 1166	53 287	0 0.6 million
North Korea	15	0	2,838	0	81	4.1 million
Iran	Not determined	0	7,534	68,947	1,058	2.1 million
Total	14,570	58.4 million	4.66 million	83,877	37,968	634.2 million
Globally	14,570	101.1 million	8.95 million	678,538	204,202	1.511 billion
Percent globally in nuclear states	100%	58%	52%	12%	19%	42%

^a^from [2]

^b^from [4]

^c^Listed as 0 in the GBD 2016 but hand search of the literature reveals the disease is endemic

So why should we care? In a previous assessment conducted in 2010, I estimated that the research and development (R&D) dollars spent on neglected diseases among the nuclear weapons countries amounted to less than 1/10,000th of the R&D funding spent to develop and maintain nuclear weapons technologies [5]. Since then, there has been only modest public health gains in terms of the reduction in the prevalence or incidence of these diseases, although measles incidence has been declining globally since the Millennium Development Goals.

As we move closer to midnight on the Doomsday Clock, the leaders of the 10 nuclear nations, which include at least six group of 20 (G20) nations where NTDs are surprisingly widespread [6], must recognize that funding and scientific activities currently focused on nuclear weapons could be redirected towards health expenditures. In so doing, the 10 nations would help to advance Sustainable Development Goals. As in 2010, I once again argue that neglected diseases are wise investments, both in the areas of implementation science and R&D.

There is little doubt that if the scientific leadership of the 10 nuclear nations were allowed to redirect their energies toward vaccine diplomacy [7], either by expanding coverage of existing vaccines and other interventions or by binational or multinational development of new vaccines and other technologies, we might see the elimination of the five diseases highlighted here. Recently, I emphasized the importance of binational cooperation between the two largest nuclear weapons nations, the US and Russia [8]. This approach led to the global elimination of smallpox, and soon polio, but it could be extended to neglected diseases [8]. Moreover, the US and Russia (as well as other nuclear nations) have troops deployed to NTD-endemic areas of the Middle East and elsewhere.

We’ve come a long way towards reducing the global prevalence and incidence of several NTDs [9]. Through vaccine diplomacy, we now have the opportunity of accelerating neglected disease elimination. In parallel, we might successfully set back the Doomsday Clock for at least a few minutes!
